# Invariant NKT Cell-Mediated Modulation of ILC1s as a Tool for Mucosal Immune Intervention

**DOI:** 10.3389/fimmu.2019.01849

**Published:** 2019-08-07

**Authors:** Stephanie Trittel, Neha Vashist, Thomas Ebensen, Benedict J. Chambers, Carlos A. Guzmán, Peggy Riese

**Affiliations:** ^1^Department of Vaccinology and Applied Microbiology, Helmholtz Centre for Infection Research, Braunschweig, Germany; ^2^Department of Medicine, Center for Infectious Medicine, Karolinska Institute, Karolinska University Hospital Huddinge, Stockholm, Sweden

**Keywords:** ILC1, iNKT cell, αGalCerMPEG, intra nasal, influenza virus, modulation

## Abstract

Non-NK group 1 innate lymphoid cells (ILC1s), mainly investigated in the mucosal areas of the intestine, are well-known to contribute to anti-parasitic and anti-bacterial immune responses. Recently, our group revealed that lung ILC1s become activated during murine influenza infection, thereby contributing to viral clearance. In this context, worldwide seasonal influenza infections often result in severe disease outbreaks leading to high morbidity and mortality. Therefore, new immune interventions are urgently needed. In contrast to NK cells, the potential of non-NK ILC1s to become functionally tailored by immune modulators to contribute to the combat against mucosal-transmitted viral pathogens has not yet been addressed. The present study aimed at assessing the potential of ILC1s to become modulated by iNKT cells activated through the CD1d agonist αGalCerMPEG. Our results demonstrate an improved functional responsiveness of murine lung and splenic ILC1s following iNKT cell stimulation by the mucosal route, as demonstrated by enhanced surface expression of TNF-related apoptosis-inducing ligand (TRAIL), CD49a and CD28, and increased secretion of IFNγ. Interestingly, iNKT cell stimulation also induced the expression of the immune checkpoint molecules GITR and CTLA-4, which represent crucial points of action for immune regulation. An *in vivo* influenza infection model revealed that intranasal activation of ILC1s by αGalCerMPEG contributed to increased viral clearance as shown by reduced viral loads in the lungs. The findings that ILC1s can become modulated by mucosally activated iNKT cells in a beneficial manner emphasize their up to now underestimated potential and renders them to be considered as targets for novel immune interventions.

## Introduction

The recent discovery of innate lymphoid cells (ILCs) expanded the pool of heterogenic innate immune cell populations. ILCs are classified into group 1, 2, and 3 ILCs according to the expression of the transcription factors T-bet, GATA-3, and RORγt, respectively. They can rapidly secrete diverse cytokines upon stimulation and were shown to represent the innate counterparts of T_H_1, T_H_2 and T_H_17 cells (1–3).

Group 1 ILCs encompass conventional NK cells as well as non-NK cells, named ILC1s. In contrast to NK cells, ILC1s express the surface receptor CD127, but lack the expression of the transcription factor Eomes (4, 5). The cytotoxic activity of ILC1s is much less prominent and mainly mediated via the expression of the TNF-related apoptosis-inducing ligand (TRAIL), whereas the killing of viral or transformed cells via inherent granule-mediated cytotoxicity is a main functional feature of NK cells next to cytokine secretion. With regard to the cytokine profile secreted upon stimulation, ILC1s primarily produce IFNγ and TNFα, similar to NK cells. Thus, ILCs expressing CD127 and T-bet but not Eomes and displaying weak cytotoxicity are considered as ILC1s.

ILCs were mainly found to be enriched at mucosal sites, where they contribute to the first line of host defense as well as to tissue homeostasis (6–8). ILC1s are also present in all secondary lymphoid tissues, albeit at very low frequencies (9). With regard to protective immunity, ILC1s were already shown to contribute to combat against bacterial as well as parasitic infections, and they were described in association with cytokine-mediated inflammation (10–13). Their impact in viral infection is less well-addressed and currently under investigation. Here, ILC1s are considered to be involved in anti-viral immunity against HIV and chronic hepatitis B (14, 15). Murine studies also suggest a role of ILC1s in cytomegalovirus and adenovirus infections (16–18). The beneficial impact of ILC1s during mucosally transmitted influenza infection as well as the importance of their cross-talk with other immune cells was recently reported by our group. In this context, differential expression of the immune checkpoint molecule GITR was shown to influence ILC1 functionality. IFNγ-secreting ILC1s displayed a differential GITR expression profile. ILC1s over-expressing GITR rather showed diminished IFNγ production. These observations were confirmed in an influenza infection model in which GITR-blocking could reverse ILC1 functionality (19). In general, GITR is described to co-stimulate T cell functionality as well as NK cell cytotoxicity (20, 21). However, its overexpression leads to ILC1 regulation in the course of influenza infection. These findings highlight its complex and differential effects depending on the cell type and immunological setting. Besides GITR, CTLA-4 represents a prominent immune checkpoint molecule that might prove useful for ILC1 modulation. CTLA-4 was shown to harbor a regulatory impact on T cells and was also described to inhibit IFNγ secretion by NK cells in response to mature DCs (22, 23). These findings also suggest an implication in ILC1 functionality.

The described features render ILC1s interesting targets for mucosal immune interventions. The development of novel formulations that act directly at the port of pathogen entry, such as influenza, are moving into the focus of vaccinology. However, despite the advances made in the field of adjuvant research, there is still a strong need for the development of mucosal immune modulators suitable for implementation in vaccine formulations. In this regard, a pegylated derivative of α-galactosylceramide (αGalCerMPEG) was demonstrated to exhibited potent mucosal adjuvant properties (24, 25). The glycolipid antigens, αGalCer and αGalCerMPEG are presented by APCs via the non-classical MHC class I molecule CD1d and recognized by invariant NKT (iNKT) cells. Upon activation, iNKT cells secrete a variety of cytokines that initiate down-stream activation of other innate and adaptive immune cells (26–28). Its strong modulating impact on inherent NK cell features upon subcutaneous (s.c.) administration further points to a possible impact on ILC1 features. However, the capacity of ILC1s to become modulated and their emerging impact on anti-viral mucosal immunity is still elusive and was therefore the aim of the present study.

## Materials and Methods

### Mice

C57BL/6 (H-2b) female mice aged 8–10 weeks were purchased from Harlan Winkelmann GmbH (now Envigo, Borchen, Germany). Jα281^−/−^ and RAG2^−/−^ mice were bred at the animal facility of the Helmholtz Center for Infection Research (HZI), Braunschweig. Mice were treated according to local and European community guidelines. They were housed under pathogen-free conditions in individual ventilated cages with food and water *ad-libitum*. The performed animal experiments were approved by the local government in Braunschweig (Germany) under the animal permission codes AZ: 33.42502-04-13/1281 and AZ: 33.19-42502-04-16/2280.

### Administration of αGalCerMPEG

The glycolipid αGalCer was pegylated at the HZI according to the published protocols (24). Briefly, αGalCer was mixed with methyl-PEG-COOH and the resulting αGalCerMPEG was purified by silica gel chromatography. Its purity was assessed by HPLC. Mice were administered with a single dose of αGalCerMPEG (5 μg (= 1.7 nmol) diluted in PBS) via the intranasal (i.n., 20 μl) or the subcutaneous (s.c., 50 μl) route. For the i.n. administration, mice were briefly anesthetized by inhalation anesthesia.

### Influenza Strains and Infection

The mouse-adapted influenza A/PR/8/34 (H1N1 PR8) strain was kindly provided by Dr. Paulina Blazejewska and Dr. Klaus Schughart (HZI). Mice were infected with a single dose containing 2^*^10^3^ foci forming units (ffu)/animal of H1N1 diluted in 20 μl PBS via the i.n. route. For this, mice were anesthetized by intra-peritoneal (i.p.) injection of ketamine/Xylazin (1 mg ketamine/0.1 mg Xylazin per 10 g body weight). The body weight was monitored on a daily basis.

### Sample Collection

Mice were euthanized and lungs, spleens and draining lymph nodes (dLNs, cervical and mediastinal) were collected and separately mashed through a 100 μm nylon strainer. Spleen and dLN homogenates were subjected to erythrocyte lysis. Lung homogenates were subsequently incubated at 37°C in 5% FCS RPMI 1640 (Life technologies, UK) containing 0.2 mg/ml collagenase D (Roche, Germany) and 20 μg/ml DNase I (Roche, Germany). Lymphocyte suspensions were purified by density gradient centrifugation (Easycoll, Biochrome GmbH, Germany). Lung and spleen lymphocytes were incubated in medium containing brefeldin A (5 μg/ml) and monensin (6 μg/ml) for 3 h at 37°C. Blood samples were collected via the retro-orbital plexus. Serum was isolated upon centrifugation and stored at −80°C until further use. Bronchoalveolar lavage (BAL) samples were collected by two intratracheal washes with 1 ml 5% FCS PBS. Serum and BAL samples were subsequently analyzed by cytometric bead array according to the manufacturer's protocol (Affymetrix/eBioscience).

### Foci Assay

The viral burden of infected mice was assessed using lung homogenates that were prepared on ice in PBS supplemented with 0.1% BSA using the Polytron 2100 homogenizer (4,000 rpm-−20 s/sample). The supernatant, cleared by centrifugation, was stored at −80°C until further use. The assay was performed as described previously (19). Briefly, serial dilutions of the lung homogenates were incubated with MDCK cells and the influenza nucleocapsid was detected by ELISA. Foci were counted under a microscope and viral titers were calculated as ffu/ml.

### Flow Cytometry Analysis

The prepared single cell suspensions were subjected to flow cytometry. Upon incubation with Fc-block (CD16/CD32, 2.4G2, Fc block, BD Biosciences), cells were stained for surface markers and subsequently fixed and permeabilized for intracellular and intranuclear staining steps according to the manufacturer's protocol (BD Bioscience, USA/Foxp3 staining kit, eBioscience, USA). The samples were processed on a FACS LSR II and Fortessa (BD Bioscience, USA) and the subsequent analysis was performed using the FlowJo software (TreeStar Inc.). ILC1s were identified according to the gating strategy published recently [(19) and Figure S1]. Briefly, living single lineage negative NKp46^+^ lymphocytes were gated on CD90^+^CD127^+^ cells and ILC1s were identified by the expression of T-bet and the lack of Eomes expression. The following antibodies were used: LIVE/DEAD Fixable Blue Dead cell stain kit (UV excitation, Invitrogen, USA), CD90.2 (53.2-1, BV785, Biolegend), CD127 (A7R34, PE-Cy5/biotin, Biolegend), NKp46 (29A1.4, A700, BD Bioscience/19A1.4, APC, eBioscience), CD3/CD19/Gr1/Ter-119 (17A2/6D5/RB6-8C5/TER-119, BV421, Biolegend), TRAIL (N2B2, biotin–streptavidin BV650, Biolegend), GITR (YGITR765, PE, Biolegend), CD28 (37.51, PerCP-Cy5.5, Biolegend), CTLA-4 (UC10-489, BV605, Biolegend; stained for surface and intracellular expression simultaneously), CD49a (Ha31/8, BV510, BD Bioscience), CD11c (N418, PE-Cy7, Biolegend), CD11b (M1/70, BV605, Biolegend), B220 (RA3-6B2, PE-Cy5, Biolegend), CD80 (16- 10A1, BV421, Biolegend), CD86 (GL-1, BV650, Biolegend), IFNγ (XMG1.2, BV711, Biolegend), T-bet (4B10, PE-Cy7, Biolegend), Eomes (Dan11mag, FITC, eBioscience), RORγt (AFKJS-9, APC, eBioscience). The t-distributed stochastic neighbor embedding (tSNE) analysis was performed using the FlowJo software (Version 10.5.3, TreeStar Inc.).

### *In vitro* Models

For assessing the impact of αGalCerMPEG on ILC1 activation *in vitro*, αGalCerMPEG-loaded bone marrow derived dendritic cells (BMDCs), sorted splenic NKT cells (B220^−^ CD11c^−^ NK1.1^+^ CD4^+^ CD8^+^ cells, sorted using a FACS Aria II cell sorter) and *in vitro*-generated ILC1s were co-cultured. ILC1s were generated from RAG2^−/−^ bone marrow cells *in vitro* as described earlier (19). NKT cells were sorted on a FACS Aria II cell sorter using the following antibodies: CD4 (GK1.5, FITC, eBioscience), CD8 (53–6.7, FITC, BD), NK1.1 (PK136, PE-Cy7, eBioscience), B220 (RA3–6B2, Pacific Blue, BioLegend), CD11c (N418, PB, BioLegend). BMDCs were generated as previously described (19). Briefly, bone marrow cells were incubated in the presence of 100 ng/ml FLT-3 ligand (Peprotech, USA) for 7–8 days. For co-culture studies these BMDCs were primed overnight with 300 ng/ml αGalCerMPEG and subsequently co-cultured in complete media supplemented with 300 ng/ml of αGalCerMPEG overnight at a ratio of 6:6:1. To address the *in vitro* impact of αGalCerMPEG on ILC1s in the context of H1N1 infection, the co-culture was set up with H1N1-infected BMDCs. For this, BMDCs were infected for 1 h with the wild type mouse-adapted H1N1 PR8 strain at a multiplicity of infection (MOI) of 1. The BMDCs were subsequently cultured for 5 h at 37°C with 5% CO_2_. After 6 h, the BMDCs were harvested, washed, counted, and used in the described co-culture experiments. For all co-culture settings, brefeldin A (5 μg/ml) and monensin (6 μg/ml) were added for the last 3 h of incubation. The phenotypic and functional analysis of the cells was performed by flow cytometry as described above.

### Statistical Analysis

The data analysis was performed using GraphPad Prism 6.0 (GraphPad Software, USA). Independent groups were statistically compared using Mann-Whitney test and for the comparison of multiple groups, the One-way ANOVA statistical analysis was applied. *P*-values ≤ 0.05 were considered statistically significant.

## Results

### Intranasal Administration of αGalCerMPEG Induces an ILC1 Activating Cytokine Milieu

**Figure 1:**
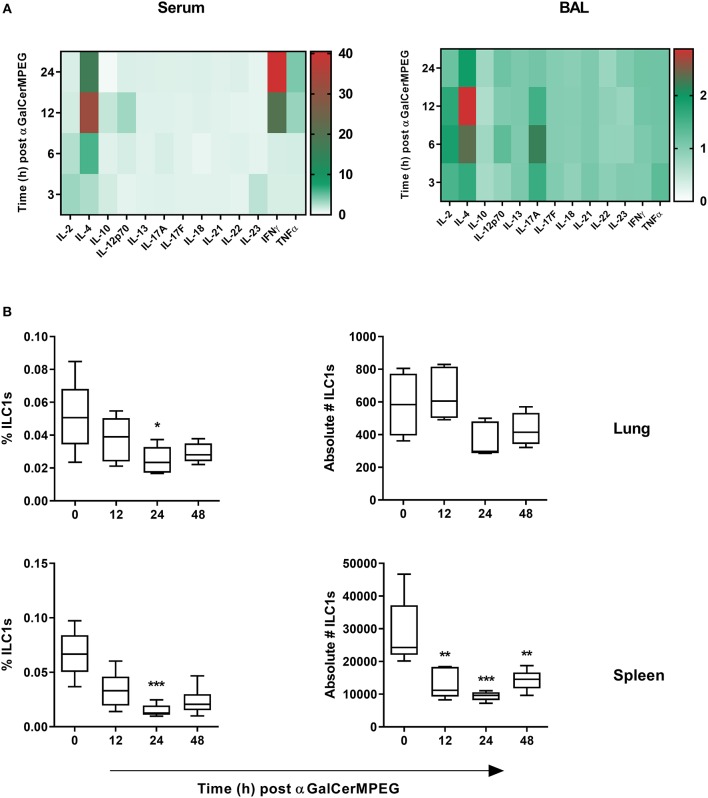
αGalCerMPEG administration induces enhanced secretion of ILC1 stimulating cytokines and reduced frequencies and absolute numbers of ILC1s. Wild type mice were administered i.n. with a single dose of αGalCerMPEG (5 μg). Sera and BAL samples were collected at the indicated time points post-administration and evaluated for their cytokine profile by CBA. **(A)** Fold change of MFI of cytokines detected in sera and BAL as compared to untreated controls. The shown data are derived from one experiment (*n* = 4). Lung and splenic lymphocytes were stained for ILC1s (CD3^−^, CD19^−^, Gr1^−^, Ter119^−^, CD90.2^+^, CD127^+^, NKp46^+^, T-bet^+^, and Eomes^−^) to perform flow cytometry analysis. **(B)** Frequencies (of total cells) and absolute numbers of lung and splenic ILC1s. Box plots represent the range in frequency and absolute number variation with the horizontal line indicating the mean. MFI and frequency data are representative from one out of two independent experiments (each with *n* = 4–5). Asterisks denote significant values calculated by One-way ANOVA as compared to untreated samples; ***p ≤ 0.001; **p ≤ 0.01; *p ≤ 0.05.

The previous finding that ILC1s contribute to the clearance of H1N1 infection via cross-talk with other immune cells renders them an interesting target for mucosal immune modulation (19). Thus, the impact of the iNKT cell-activating CD1d agonist αGalCerMPEG on ILC1 functionality was investigated. Wild type mice received a single dose of αGalCerMPEG (5 μg/animal) by i.n. route to gain insight into its impact on the mucosal and systemic cytokine environment. At different time points, serum and BAL samples were analyzed with regards to changes in the concentration of various cytokines known to impact adaptive as well as innate immune cell populations. After 6 h an increased expression of IL-4 was detected in serum as well as BAL samples, peaking 12 h after administration. In serum and BAL samples, αGalCerMPEG administration induced increased levels of IL-12 as early as 12 and 6 h after treatment, respectively. After 12 and 24 h enhanced IFNγ levels were observed in serum but not in BAL samples (Figure 1A).

To investigate whether αGalCerMPEG-induced changes in the cytokine milieu influence ILC1s, lung and spleen derived lymphocytes were analyzed regarding their frequencies and absolute numbers by flow cytometry (Figure S1). After 24 h significantly decreased frequencies of splenic and lung-derived ILC1s were observed (Figure 1B). The absolute cell numbers of lung ILC1s were marginally decreased 24 and 48 h post-administration, whereas splenic ILC1 numbers were significantly decreased at all investigated time points after αGalCerMPEG administration. The obtained data show that the administration of αGalCerMPEG supports the generation of an ILC1-activating cytokine environment while resulting in reduced frequencies and absolute numbers of ILC1s at both local and systemic levels.

### Intranasal Administration of αGalCerMPEG Results in Enhanced ILC1 Activation

**Figure 2:**
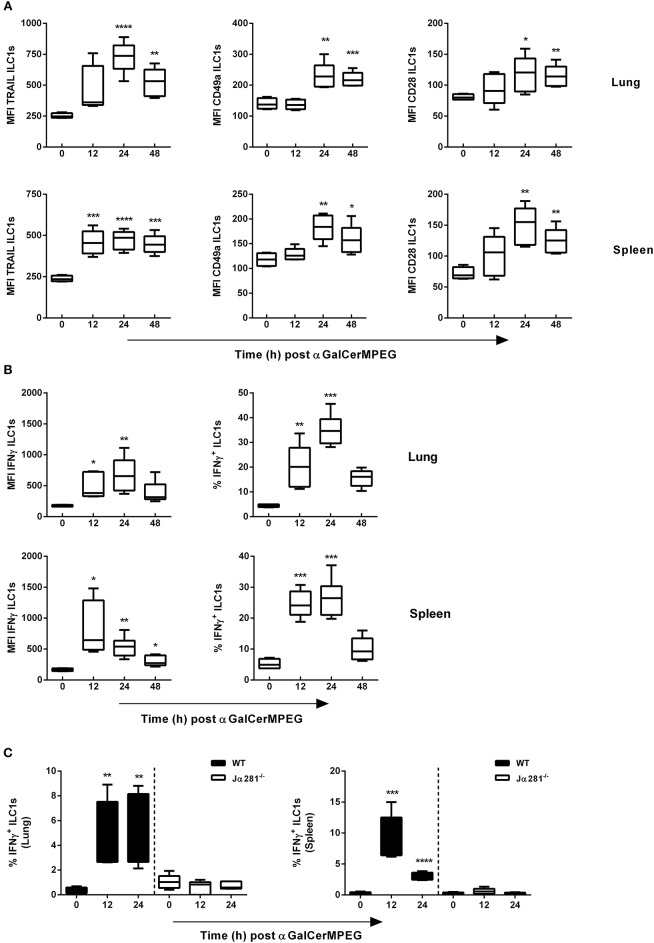
αGalCerMPEG treatment results in activation of ILC1s. Wild type (WT) and Jα281^−/−^ mice received a single dose of αGalCerMPEG (5 μg) by i.n. route. Subsequently, lung and splenic lymphocytes were stained for the surface activation markers TRAIL, CD49a and CD28 as well as IFNγ, and analyzed by flow cytometry upon 3 h incubation in the presence of monensin and brefeldin. **(A)** MFI of lung and splenic ILC1s expressing TRAIL, CD49a, and CD28. **(B)** MFI and frequency (of ILC1s) of IFNγ production by lung and splenic ILC1s. **(C)** Frequencies (of ILC1s) of lung and splenic IFNγ^+^ ILC1s derived from wild type and Jα281^−/−^ mice. Box plots represent the range in MFI variation with the horizontal line indicating the mean. MFI data are representative from one out of two independent experiments (each with *n* = 4–6) for **(A)** and **(B)** and from one experiment (*n* = 4–6) for **(C)**. Asterisks denote significant values calculated by One-way ANOVA as compared with untreated samples; ****p ≤ 0.0001; ***p ≤ 0.001; **p ≤ 0.01; *p ≤ 0.05.

The observed impact of αGalCerMPEG on ILC1 frequencies and numbers prompted the functional evaluation of lung and splenic ILC1s. Therefore, following i.n administration of αGalCerMPEG, surface markers known to be expressed following ILC1 activation were evaluated at different time points. A significantly enhanced expression of TRAIL on lung ILC1s was observed 24 and 48 h after administration, whereas splenic ILC1 showed a significantly elevated expression at all analyzed time points (Figure 2A). The administration of αGalCerMPEG resulted in elevated expression of CD49a and CD28 after 24 and 48 h in both organs as compared to untreated controls (Figure 2A). The analysis of IFNγ secretion revealed significantly increased expression densities (MFI) as well as frequencies in the lung after 12 and 24 h (Figure 2B). The frequency of splenic IFNγ-secreting ILC1s was enhanced 24 and 48 h after administration of αGalCerMPEG and significantly increased expression densities were detected at all analyzed time points. These results demonstrate that ILC1s at mucosal as well as lymphoid tissues can be phenotypically and functionally modulated by administration of the glycolipid αGalCerMPEG. The stimulation of iNKT cells via the s.c. route resulted in a similar activation of lung and splenic ILC1s as observed for the i.n. route (Figure S2).

The iNKT cell-dependent activation of different populations of the innate (e.g., NK cell and DCs) as well as the adaptive (e.g., CD4^+^ and CD8^+^ T cells) immune system by αGalCerMPEG was already shown Ebensen et al. (24). However, the importance of iNKT cells for the observed αGalCerMPEG-induced ILC1 activation is still elusive. Therefore, iNKT cell-deficient mice (Jα281^−/−^) received αGalCerMPEG by i.n. route and the secretion of IFNγ by ILC1s was assessed. The lack of iNKT cells completely abolished αGalCerMPEG-induced enhanced IFNγ expression and the increased frequencies of IFNγ^+^ ILC1s observed in the lungs and spleen of wild type mice (Figure 2C and Figure S3). These findings clearly show the iNKT cell dependency of the detected αGalCerMPEG-induced ILC1 activation after mucosal application.

### αGalCerMPEG-Activated iNKT Cells Stimulate GITR^+^ But Not GITR^−^ ILC1s

**Figure 3:**
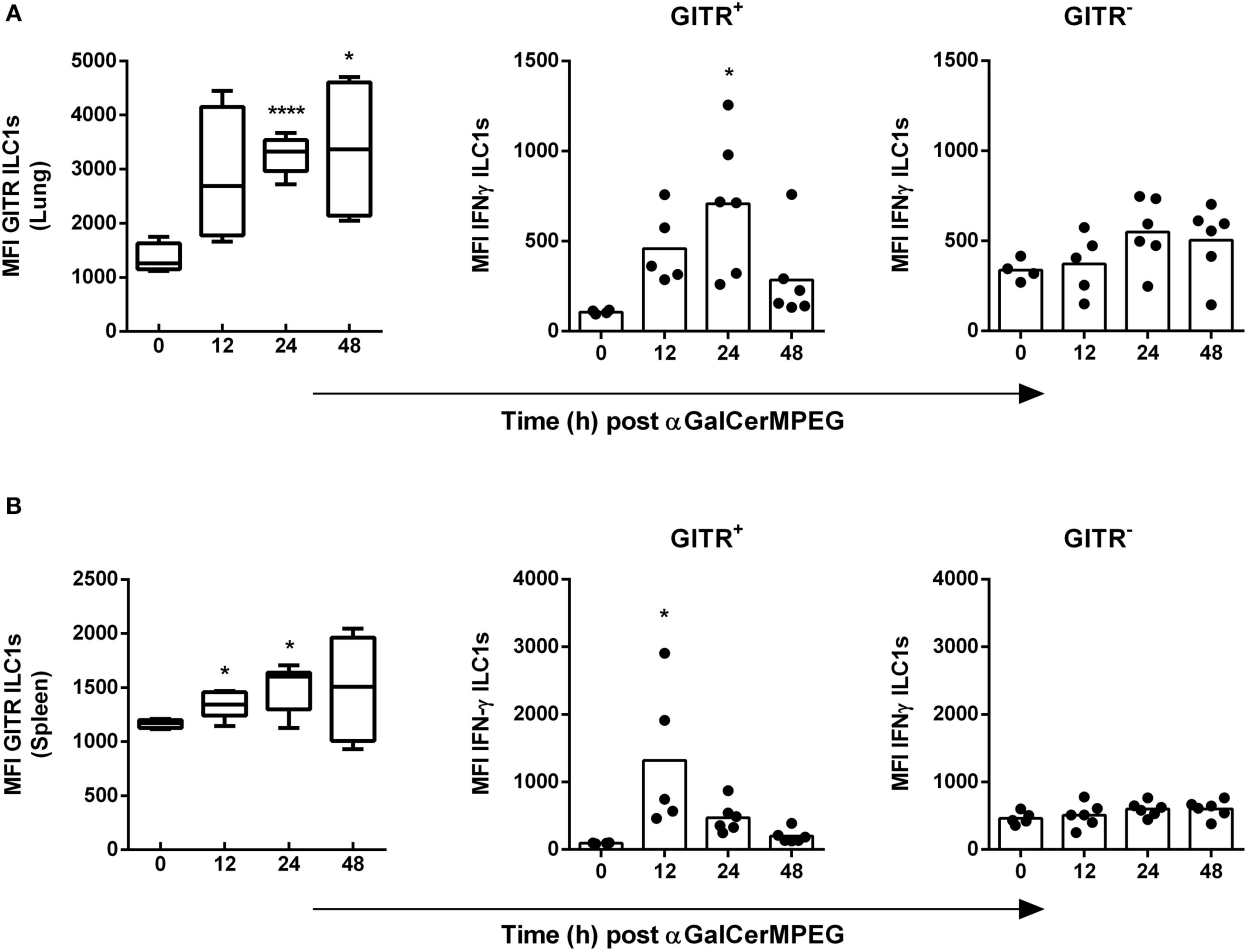
Enhanced IFNγ secretion by GITR^+^ ILC1s following i.n. treatment with αGalCerMPEG. Wild type mice were administered i.n. with a single dose of αGalCerMPEG (5 μg). Lung and splenic lymphocytes were stained for GITR and IFNγ expression by ILC1s and subjected to flow cytometry analysis. MFI of GITR on ILC1s and MFI of IFNγ by GITR^+^ and GITR^−^ ILC1s derived from **(A)** lung and **(B)** spleen. Box plots represent the range in MFI with the horizontal line indicating the mean. MFI data are representative from one out of two independent experiments (each with *n* = 4–6). Asterisks denote significant values calculated by One-way ANOVA and compared with untreated samples; ****p ≤ 0.0001*; *p* ≤ 0.05.

We recently showed in a murine influenza infection model that the differential expression of GITR, a stimulatory immune checkpoint molecule, can influence the activation status of ILC1s. Here, we assessed whether the level of GITR expression by ILC1s and subsequently their functional responsiveness can be modulated by i.n. administered αGalCerMPEG. Significantly enhanced expression densities of GITR on lung ILC1s were observed 24 and 48 h after iNKT cell stimulation (Figure 3A). The increase in GITR expression was accompanied by elevated IFNγ secretion by GITR^+^ but not GITR^−^ ILC1s, which peaked 24 h after iNKT cell stimulation. For splenic ILC1s, increased expression levels of GITR were detected 12 and 24 h after i.n. αGalCerMPEG administration. Similar to lung ILC1s, splenic GITR^+^ ILC1s showed significantly enhanced IFNγ secretion after 12 h whereas no changes were observed for GITR^−^ ILC1s as compared to untreated controls (Figure 3B). The obtained finding reveals αGalCerMPEG-induced changes on the GITR expression level and enhanced cytokine secretion of GITR^+^ ILC1 subsets.

### ILC1 Stimulation by αGalCerMPEG Is Characterized by an Enhanced CTLA-4 Expression

**Figure 4:**
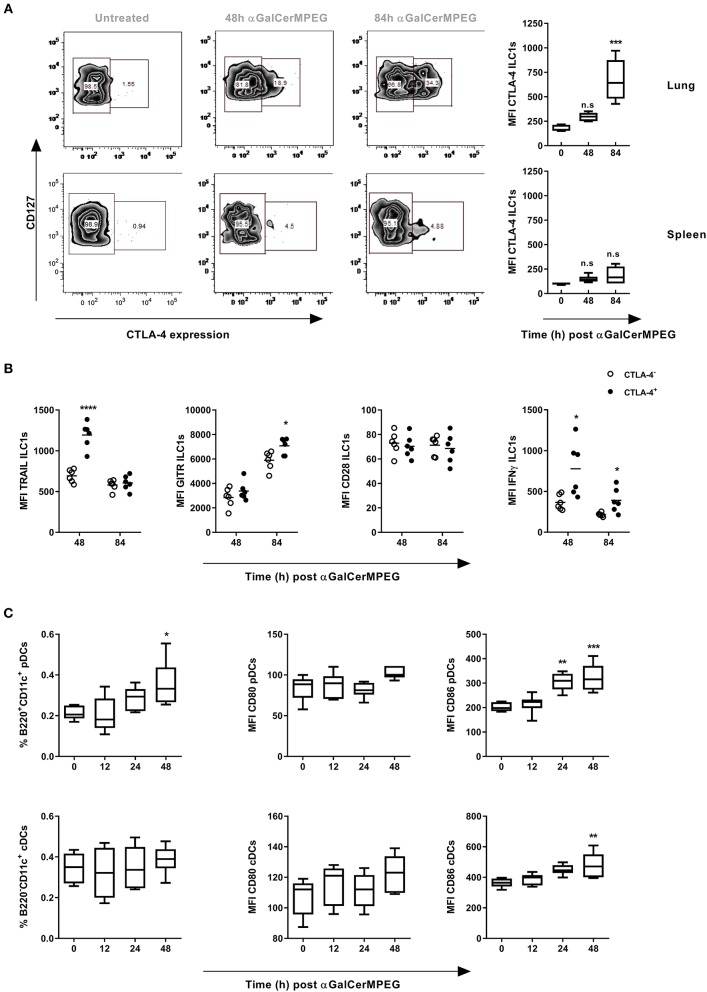
αGalCerMPEG-induced enhanced functionality of lung ILC1s is associated with CTLA-4 expression. Wild type mice were treated i.n. with a single dose of αGalCerMPEG (5 μg). Lung and splenic lymphocytes were isolated 48 and 84 h after administration and stained for ILC1 expression of CTLA-4 and activation markers (TRAIL, GITR, CD28, IFNγ) for the flow cytometric analysis upon 3 h of incubation in the presence of monensin and brefeldin. **(A)** Representative dot plots show the expression of CTLA-4 on lung and splenic ILC1s. Diagrams show the expression density (MFI) of CTLA-4 by lung and splenic ILC1s. **(B)** MFI of TRAIL, GITR, CD28, and IFNγ by lung CTLA-4^+^ and CTLA-4^−^ ILC1s. DCs from dLNs (mediastinal and cervical) were processed for flow cytometry analysis and stained for markers related to pDC (B220^+^CD11c^+^) and cDC (B220^−^CD11c^+^) identification and maturation. **(C)** Frequencies (of living singlet cells) of DC subsets and MFI of CD80 and CD86. Box plots represent the range in MFI with the horizontal line indicating the mean. Scatter plots represent the range in MFI with the horizontal line indicating the mean. MFI and frequencies from one experiment are shown (*n* = 4–6). Asterisks denote significant values calculated by One-way ANOVA as compared to untreated samples **(A)** and **(C)** or by unpaired, two-tailed Student's *t*-test or comparing CTLA-4^−^ and CTLA-4^+^ **(B)**; ****p ≤ 0.0001; ***p ≤ 0.001; **p ≤ 0.01; *p ≤ 0.05.

Next to GITR, CTLA-4 represents an interesting stimulatory immune checkpoint molecule which is described to control T cell and NK cell activation. Thus, surface and intracellular CTLA-4 expression by lung and splenic ILC1s following i.n. administration of αGalCerMPEG was assessed. The stimulation of iNKT cells resulted in enhanced frequencies of lung CTLA-4^+^ ILC1s as well as significantly increased expression of CTLA-4 after 48 and 84 h as well as 84 h, respectively (Figure 4A). In contrast, splenic ILC1s did not show meaningful changes in frequency or expression density with regard to CTLA-4. The αGalCerMPEG-induced up-regulation of CTLA-4 *in vivo* was further confirmed in an *in vitro* co-culture of BMDCs, sorted NKT cells and *in vitro*-generated ILC1s (Figure S4A).

Subsequently, CLTA-4^+^ and CTLA-4^−^ lung ILC1s were compared with regard to their surface expression of activation markers as well as the secretion of cytokines to assess the impact of CTLA-4 expression on ILC1 functionality. Strikingly, significantly enhanced expression densities of TRAIL and IFNγ were observed after 48, and 48 as well as 84 h, respectively, which correlate with an elevated expression of CTLA-4 (Figure 4B). Furthermore, enhanced GITR expression was observed in the CTLA-4^+^ but not CTLA-4^−^ ILC1 subsets 84 h after αGalCerMPEG administration. The expression of CD28 was not associated with CTLA-4 expression. These results demonstrate that αGalCerMPEG-mediated activation of ILC1s induces the expression of CTLA-4 on ILC1s. In addition, the observed correlation of CTLA-4 with enhanced expression of the activation markers TRAIL and GITR, and the secretion of IFNγ reveals that only activated lung ILC1s express CTLA-4. These findings were consistent with conducted *in vitro* co-culture experiments of BMDCs, sorted NKT cells and *in vitro* generated ILC1s, in which the αGalCerMPEG-induced secretion of IFNγ was restricted to the CTLA-4^+^ ILC1 subset (Figures S4A,B).

Subsequently, it was investigated whether αGalCerMPEG induces changes in the expression patterns of CTLA-4 ligands on DCs. To this end, cells isolated from dLNs (mediastinal and cervical) of αGalCerMPEG-treated mice were analyzed with regard to the frequencies of different DC subsets and the expression of the CTLA-4 ligands CD80 and CD86. Significantly increased frequencies of pDCs were observed 48 h after treatment (Figure 4C). Furthermore, iNKT cell activation induced enhanced expression of the CTLA-4 ligand CD86 24 and 48 h later, whereas no impact on CD80 was observed. Interestingly, the frequency of cDCs was not affected by αGalCerMPEG, but the expression of CD86 by cDCs was significantly increased after 48 h. The assessment of pulmonary DCs (MHC cl. II^+^CD11c^+^) revealed significantly enhanced frequencies as well as marginally increased expression of CD80 and CD86 upon i.n. administration of αGalCerMPEG (Figure S4C). The obtained data suggest an interaction of ILC1s with different DC subsets via the ligation of CTLA-4 to their respective ligands upon αGalCerMPEG-stimulation of iNKT cells.

### Stimulation of iNKT Cells Activates ILC1s in the Course of Influenza Infection

**Figure 5:**
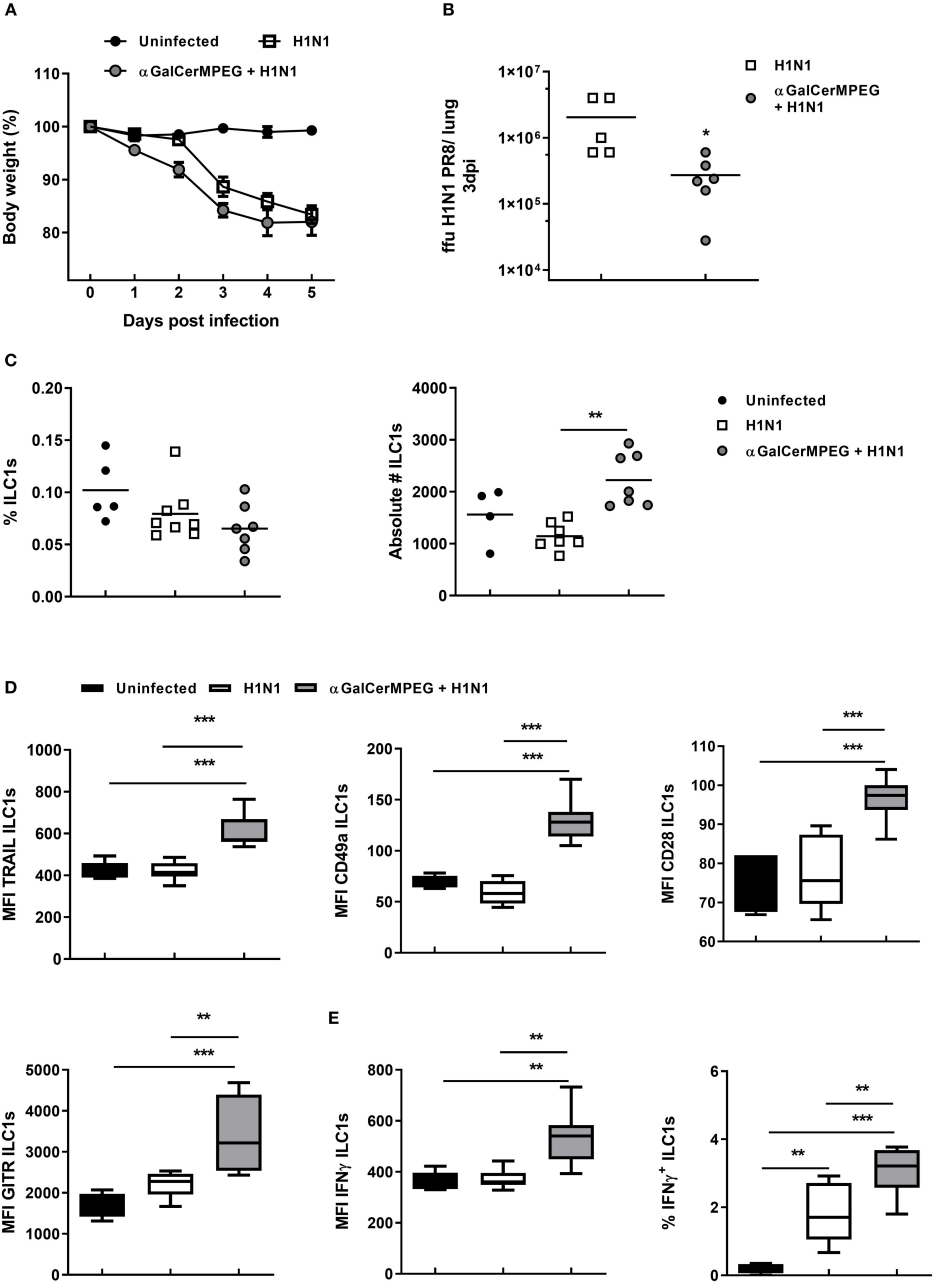
H1N1 infection of αGalCerMPEG-treated mice induced ILC1 activation. Wild type mice were administered i.n. with a single dose of αGalCerMPEG (5 μg) 12 h prior to H1N1 infection (2*10^3^ ffu). **(A)** Changes in body weight (%) post-infection. **(B)** Lung viral burden as determined 3 days post-infection by foci assay. Scatter plots represent viral loads as ffu per lung. Depicted data are representative from one out of two independent experiments (each with *n* = 5–7). Lung lymphocytes were isolated 3 days post-infection and analyzed for **(C)** frequencies and absolute numbers of ILC1s and **(D)** their expression density (MFI) of the activation markers TRAIL, CD49a, CD28, and GITR by flow cytometry analysis. Frequency and absolute cell number data are representative from one out of two independent experiments (each with *n* = 5–7). MFI data for the expression of the activation markers are shown from one experiment (*n* = 5–7). **(E)** Lung lymphocytes secreting IFNγ (MFI and frequencies) analyzed by flow cytometry following 3 h incubation in media with monensin and brefeldin. MFI and frequency data are representative from one out of two independent experiments (each with *n* = 5–7). Box plots represent the range in frequency variation as well as absolute cell number with the horizontal line indicating the mean. Asterisks denote significant values calculated by One-way ANOVA; ***p ≤ 0.001; **p ≤ 0.01; *p ≤ 0.05.

Our group showed that ILC1s contribute to the clearance of influenza infections. Therefore, the observed phenotypic and functional modulation of ILC1s by αGalCerMPEG-activated iNKT cells raised the question of whether this approach can be used to improve protective immunity against influenza infections. In order to address this point, mice which received αGalCerMPEG i.n. and were infected 12 h later with influenza by the natural i.n. route. Mice treated with αGalCerMPEG as well as untreated but infected mice showed a body weight reduction starting from 2 to 3 days post-infection, respectively (Figure 5A). The viral burden in the lungs assessed 3 days post-infection revealed that pre-activation of iNKT cells caused a significant reduction of lung viral titers as compared to untreated influenza-infected mice (Figure 5B). These findings demonstrate that αGalCerMPEG boosts protective immune responses toward influenza infection. In order to assess the impact of αGalCerMPEG on ILC1s in the course of influenza infection, changes in their phenotype and functional responsiveness were analyzed. Flow cytometry analysis of lung- and spleen-derived lymphocytes was carried out 3 days post-infection. Influenza infection alone resulted in reduced frequencies of lung ILC1s which was further strengthened by prior iNKT cell activation. The absolute number of lung ILC1s was not affected as compared to uninfected controls, but was significantly increased upon pre-treatment with αGalCerMPEG as compared to infected untreated mice (Figure 5C).

A significant drop in ILC1 frequencies and absolute numbers was detected in the spleen after infection as compared to uninfected controls (Figure S5A). Pre-treatment with αGalCerMPEG prior to infection could reverse this effect and resulted in significantly increased numbers of splenic ILC1s as compared to untreated but infected groups.

These results show that stimulated iNKT cells impact ILC1s and thus prompted the evaluation of a functional ILC1 modulation in the course of influenza infection. A significantly up-regulated expression of TRAIL and CD49a was observed on lung ILC1s derived from pre-treated influenza-infected mice as compared to the untreated infected and uninfected control groups (Figure 5D). For splenic ILC1s, increased expression densities of TRAIL and CD49a in the αGalCerMPEG-pre-treated infected group were observed as compared to the non-treated infected group (Figure S5B). Significantly enhanced GITR expression was also detected on splenic ILC1s upon influenza infection, whereas in the lung only minor changes were observed (Figure 5D and Figure S5B). The pre-treatment with αGalCerMPEG, however, resulted in an additionally increased GITR expression on ILC1s in both organs. With regard to CD28, an enhanced expression was observed on lung ILC1s derived from αGalCerMPEG pre-treated infected mice as compared to the uninfected as well as infected but untreated groups (Figure 5D). With regard to splenic ILC1s, influenza infection alone resulted in an up-regulated expression of CD28 that was not further boosted by the pre-activation of iNKT cells (Figure S5B).

These data show that αGalCerMPEG administration significantly alters the phenotype of ILC1s in the course of infection locally, at the site of administration and infection, as well as at systemic level.

In order to assess whether the phenotypic activation is accompanied by functional changes, lung and splenic ILC1s were subsequently assessed for their cytokine secretion capacity 3 days post-infection. The expression density of IFNγ by lung and splenic ILC1s was not affected by the infection alone as compared to uninfected controls. Pre-treatment with αGalCerMPEG significantly boosted the IFNγ expression density of lung and splenic ILC1s as compared to uninfected or untreated infected controls (Figure 5E and Figure S5C). The frequencies of IFNγ^+^ lung ILC1s were significantly elevated upon infection, whereas splenic IFNγ^+^ ILC1s were only marginally increased (Figure 5E and Figure S5C). The pre-activation of iNKT cells by αGalCerMPEG prior to the infection resulted in even higher frequencies of IFNγ^+^ lung ILC1s, whereas only a minor additional effect was observed for spleen-derived ILC1s. The presented data prove that αGalCerMPEG can modify both local and systemic ILC1 responses during influenza infection. Furthermore, the data clearly demonstrate the capacity of αGalCerMPEG for boosting and fine-tuning ILC1 functionality in the course of an H1N1 influenza infection.

### αGalCerMPEG-Activated ILC1s Expressing CTLA-4 Display Increased Functionality in Course of Influenza Infection

**Figure 6:**
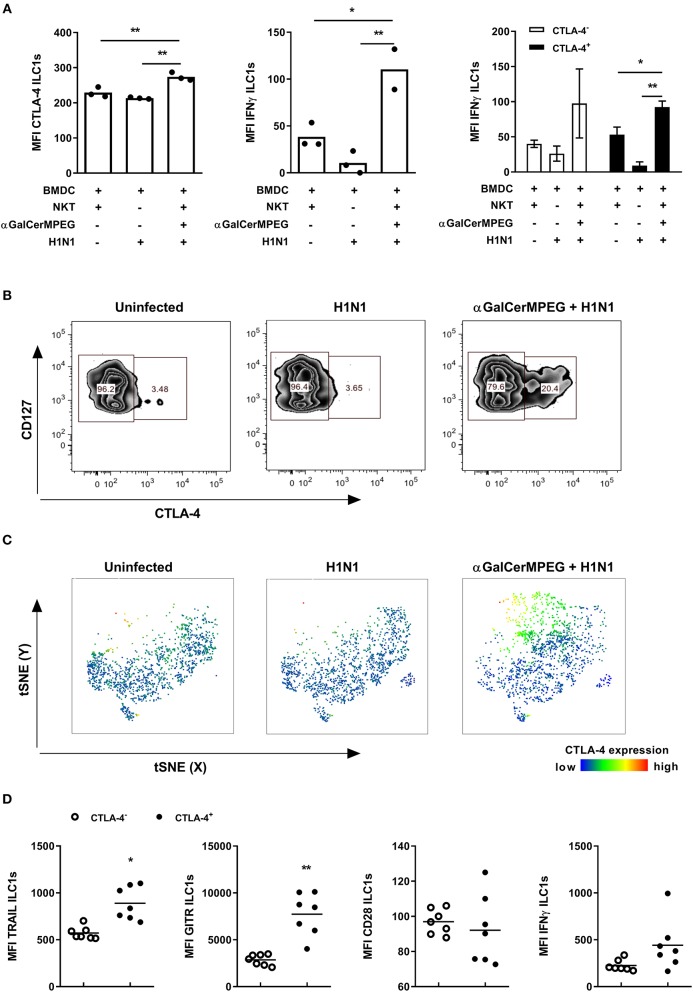
αGalCerMPEG-induced CTLA-4 expression correlates with αGalCerMPEG-activated ILC1s. FLT-3L differentiated BMDCs were first treated overnight with αGalCerMPEG (300 ng/ml) and subsequently BMDCs were infected with H1N1 (MOI 1). BMDCs treated with αGalCerMPEG were co-cultured with *in vitro*-generated ILC1s and sorted splenic NKT cells overnight at a 6:6:1 ratio. ILC1s were assessed for the expression of CTLA-4 and IFNγ by flow cytometry following 3 h incubation in the presence of monensin and brefeldin. **(A)** MFI of ILC1s expressing CTLA-4 and IFNγ and MFI of IFNγ expression by CTLA-4^+^ and CTLA-4^−^ ILC1s. Bars represent mean ± SEM. MFI data are shown from one experiment (*n* = 3–4 technical replicates). Wild type mice were injected i.n. with a single dose of αGalCerMPEG (5 μg) 12 h prior to H1N1 infection (2*10^3^ ffu). Lung lymphocytes were analyzed by flow cytometry 3 days post-infection with regard to CTLA-4 expression and markers related to ILC1 functionality following 3 h incubation in the presence of monensin and brefeldin. **(B)** Representative dot plots depicting CTLA-4 expression by lung ILC1s. **(C)** tSNE analysis of ILC1s indicating the expression density of CTLA-4. **(D)** Expression densities of TRAIL, GITR, CD28, and IFNγ by CTLA-4^+^ and CTLA-4^−^ lung ILC1s isolated from αGalCerMPEG-treated and influenza-infected mice. Box plots and scatter plots represent the range in MFI with the horizontal line indicating the mean. MFI data are displayed from one experiment (*n* = 5–8). Asterisks denote significant values as calculated by unpaired student's *t*-test or by One-way ANOVA; **p ≤ 0.01; *p ≤ 0.05.

The observed impact of activated iNKT cells on the phenotype and functionality of lung and splenic ILC1s as well as the implication of CTLA-4 expression for ILC1 responsiveness also raised the question of whether CTLA-4 expression by ILC1s is affected in the course of influenza infection. To approach this assumption, the expression of CTLA-4 and IFNγ by ILC1s was assessed in an *in vitro* co-culture system comprising H1N1-infected BMDCs, NKT cells and *in vitro* generated ILC1s as previously described (19). A significantly enhanced expression density of CTLA-4 and IFNγ by ILC1s was observed when NKT cells and ILC1s were co-cultured with influenza-infected BMDCs in the presence of αGalCerMPEG as compared to co-cultures of H1N1-infected BMDCs in the absence of NKT cell stimulation or uninfected BMDCs and unstimulated NKT cells (Figure 6A). The functional analysis of ILC1s with regard to CTLA expression identified CTLA-4^+^ ILC1s as the main producers of IFNγ in this *in vitro* setting.

*In vivo*, the i.n. administration of αGalCerMPEG resulted in an up-regulation of CTLA-4 on lung ILC1s. A beneficial role of memory T cell responses in the course of influenza infection was recently ascribed to the interaction of CTLA-4 with its corresponding ligands CD80/CD86 (29). Thus, to assess the impact of H1N1 infection on CTLA-4 expression, lung ILC1s were analyzed 3 days post-infection. Influenza infection alone did not impact the expression density or frequencies of CTLA-4^+^ ILC1s as compared to uninfected controls, whereas prior iNKT cell stimulation by αGalCerMPEG induced significantly enhanced frequencies of CTLA-4^+^ ILC1s as well as increased expression density levels (Figures 6B,C). To further assess the impact of CTLA-4 expression on ILC1 functionality, CTLA-4^+^ and CTLA-4^−^ ILC1s derived from αGalCerMPEG-treated H1N1-infected mice were analyzed 3 days post-infection. CTLA-4^+^ lung ILC1s showed enhanced expression densities of TRAIL and GITR as compared to CTLA-4^−^ ILC1s, whereas the expression of CD28 did not differ between both populations (Figure 6D). The production of IFNγ was marginally increased in the CTLA-4-expressing ILC1 subset. These data confirm a link between the expression of CTLA-4 and the functionality of ILC1s upon mucosal stimulation with αGalCerMPEG in the course of influenza infection.

## Discussion

Our previously published studies demonstrated that ILC1s distinctly contribute to the clearance of influenza infection, partly by cross-talking with innate and adaptive immune cells (19). Here, an influenza-induced activation of ILC1s was shown by enhanced expression of different surface activation markers and secreted cytokines. These findings prompted the question of whether ILC1s can be modulated to promote anti-viral immunity. To address this issue, the impact of a pegylated derivative of the iNKT cell agonist αGalCer on ILC1 functionality was investigated. This compound was already shown to affect various innate and adaptive immune cells populations (24, 25, 30). In this context, αGalCerMPEG-activated iNKT cells were reported to modulate inherent NK cell features, thereby impacting anti-viral responses in a murine cytomegalovirus infection model (31). The close similarity of NK cells and ILC1s cells, together with the mucosal adjuvant properties of αGalCerMPEG (24, 32) suggest that it might also be a promising modulator of ILC1 functionality when administered by mucosal route.

The i.n. treatment of wild type mice with αGalCerMPEG resulted in enhanced levels of a variety of cytokines including the iNKT cell-characteristic cytokine IL-4 as well as IL-12 and IFNγ with differential patterns observed in serum and BAL samples. These findings indicate the promotion of T_H_1 as well as T_H_2 responses thus indicating a balanced adaptive immune response. Stimulation of iNKT cells with αGalCerMPEG resulted in the activation of ILC1s as shown by the increased expression of different activation markers as well as an enhanced cytokine production. Interestingly, this activation was not limited to the lung, but was also observed for splenic ILC1s. The observed increased expression of TRAIL correlates with an enhanced cytotoxic potential. In this regard, a previous report showed that stimulation of NKT cells with αGalCer induced an upregulated TRAIL expression by liver NK cells (33). Furthermore, TRAIL was reported to contribute to immunity against HIV, hepatitis, CMV and influenza amongst others (34, 35). The induction of TRAIL expression by ILC1s might thus directly contribute to anti-viral immunity. An enhanced activation status of ILC1s upon administration of αGalCerMPEG is further reflected by an elevated expression of CD49a. This is in line with earlier findings showing a correlation of enhanced CD49a expression and elevated functionality by T cells and NKT cells upon *in vivo* stimulation with concanavalin-A (36). CD49a was further described as a ligand implicated in retaining immune cells within tissues (37). Thus, besides its function as an activation marker, CD49a might promote the retention of activated ILC1s within the lung, thereby supporting local immune responses. CD28 expression was also found to be enhanced on ILC1s following αGalCerMPEG stimulation by the mucosal route. CD28 was reported to induce the proliferation of murine IL-2-activated NK cells *in vitro* and enhance their IFNγ-production via the interaction with its ligands CD80 and CD86 (38). Thus, the findings of increased expression of TRAIL, CD49a, and CD28 corroborates that αGalCerMPEG administered i.n. promotes the activation of ILC1s. Furthermore, the stimulation of iNKT cells resulted in increased IFNγ secretion by ILC1s. Interestingly, a previous study reported that CD49a expression on T cells drives IFNγ production, thereby promoting inflammation (36). An additional study showed that protection against *Toxoplasma gondii* infection could be mediated by CD28^+^ NK cells displaying increased IFNγ production and cytotoxicity (39). A similar mechanism might be triggered in ILC1s following stimulation with αGalCerMPEG. The increased IFNγ secretion might be further elicited by the observed αGalCerMPEG-induced upregulation of IL-12 and IFNγ. These cytokines were described to play crucial roles in promoting IFNγ production by NK cells (40, 41). Treatment of Jα281^−/−^ mice, which lack NKT cells, with αGalCerMPEG did not result in any changes with regard to the activation status and functionality of ILC1s as compared to untreated mice. This is consistent with recent studies from our group demonstrating that the αGalCerMPEG-mediated activation of NK cells is crucially dependent on the presence of iNKT cells as well as CD1d expression (24, 31). The s.c. administration of αGalCerMPEG showed the same stimulatory impact on lung and splenic ILC1s as the i.n. administration thus highlighting the potential of immune cell stimulation via the i.n. route.

Assessing the potential role of αGalCerMPEG-activated ILC1s in the course of H1N1 infection *in vivo* revealed reduced viral titers in mice pre-treated with αGalCerMPEG. The increase in the absolute numbers of lung ILC1s correlated with an increased expression of TRAIL, CD49a, and CD28 in mice treated with αGalCerMPEG prior to influenza infection. This suggests that pre-activated ILC1s exhibit a higher responsiveness, thereby contributing to viral clearance. The elevated CD28 levels on lung ILC1s indicate a potential role for the CD28-CD80/CD86 axis leading to improved ILC1s responsiveness during influenza infection. This is in line with a study describing a crucial impact of the CD28-CD80/CD86 axis for anti-viral CD8^+^ T cell responses (42). Furthermore, an increased IFNγ secretion by lung ILC1s was observed in αGalCerMPEG-pre-treated and infected mice. Secretion of IFNγ downstream of iNKT cell activation was previously shown to enhance the cytolytic activities of NK as well as virus-specific CD8^+^ T cells, resulting in reduced influenza viral burden and enhanced survival (43). Similarly, stimulation of iNKT cells improved the disease outcome after influenza infection by boosting innate responses mediated by NK cells (44). Thus, the obtained results clearly indicate that stimulation of iNKT cells using αGalCerMPEG boosts the functionality of lung ILC1s, which might in turn contribute to early anti-viral immunity.

Next to the expression of activation markers and cytokine secretion, the impact of αGalCerMPEG-activated iNKT cells on the expression of the immune checkpoint molecules GITR and CTLA-4 by ILC1s was investigated. GITR signaling was already shown to inhibit regulatory T cells, thereby supporting the balance between effector and regulatory T cells and promoting CD4^+^ and CD8^+^ T cell responses (45–47). Recently, GITR was identified as a novel mechanism regulating ILC1 functionality *in vivo* in the course of influenza infection as well as *in vitro* upon cytokine stimulation (19). However, an impact of iNKT cell activation on GITR expression by ILC1s has not been reported so far. Interestingly, the activation of ILC1s by αGalCerMPEG-stimulated iNKT cells resulted in increased GITR expression with GITR^+^ ILC1s displaying enhanced functionality. GITR up-regulation on memory CD8^+^ T cells was previously demonstrated to result in an increased survival in the bone marrow dependent on IL-15 (48). Interestingly, elevated levels of the cytokine IL-15 were also observed in sera of αGalCerMPEG-treated mice (31). Thus, αGalCerMPEG-induced iNKT cell stimulation might boost the functional response of ILC1s by promoting their survival. The observation of increased GITR-L expression on DCs hints toward a potential interaction with GITR^+^ ILC1s following iNKT cell stimulation (data not shown). In the influenza infection model, αGalCerMPEG-pre-treated mice showed an enhanced GITR expression on ILC1s as compared to infected untreated controls. This interaction might subsequently boost the potential of DCs to present antigens thus supporting enhanced anti-viral immunity. On the other hand, the elevated expression of GITR could also serve as a built-in safety feature to control immune activation. The ligation of GITR expressed on iNKT cells by an agonistic anti-GITR mAb was shown to negatively regulate their proliferation and cytokine secretion in response to αGalCer (49). Here, αGalCerMPEG-stimulated iNKT cells might initially trigger ILC1 activation but also promote GITR-upregulation thereby keeping the balance of immune activation and preventing overwhelming immune responses.

Besides the up-regulated expression of GITR on ILCs, iNKT cell stimulation by αGalCerMPEG induced an increased CTLA-4 expression on ILC1s over time. CTLA-4 has been extensively studied with regard to T cell biology and its role as an immune checkpoint molecule rendered it an attractive target for immune therapy (50–52). In this regard, the manipulation of CTLA-4 signaling (e.g., by using Ipilimumab) has been investigated in clinical trials and approved for treatment of advanced melanoma (53–55). The increased αGalCerMPEG-induced expression of the co-stimulatory receptor CD86 on dLN-derived DCs hints toward a potential interaction of CTLA-4^+^ ILC1s with CD86^+^ DCs. Interestingly, CTLA-4^+^ ILC1s displayed a higher activation status at earlier time points. This observation concurs with T cell data showing high CTLA-4 expression on activated, proliferating T cells 24–72 h after stimulation (56). Enhanced CTLA-4 expression at later time points after iNKT cell stimulation was associated with a gradual decrease of responsiveness, as demonstrated by reduced IFNγ secretion and TRAIL expression and increased GITR expression. These findings suggest a regulatory function for CTLA-4 expressed by ILC1s. In this line, activated NK cells expressing CTLA-4 were described to display reduced IFNγ production in response to interaction with mature DCs (23). The mechanism of functional immune regulation might be explained by competition with the co-stimulatory receptor CD28 for their shared ligands CD80 and CD86 expressed by APCs. Compared to the constitutive expression of CD28, CTLA-4 is known to be expressed by activated immune cells at later time points exhibiting higher ligand affinity. Thus, CTLA-4 expression on ILC1s might compete with CD28, thereby regulating the functional responsiveness of iNKT cell-modulated ILC1s. However, further studies are required to confirm a direct regulatory impact of CTLA-4 on ILC1 functionality and ascertain the mode of action of CTLA-4-induced regulation of ILC1 responses. The observation of αGalCerMPEG-induced immune-modulation of ILC1s resulting in enhanced functionality suggest a beneficial effect on the anti-viral immune responses of ILC1s against influenza infection. However, immune stimulation requires to be regulated to prevent over-shooting responses that might lead to morbidity and mortality (57, 58). In this regard, the simultaneously enhanced ILC1-mediated anti-viral responses and subsequent upregulation of CTLA-4 and GITR expression on lung ILC1s highlight the potential of αGalCerMPEG as a novel immune-modulator which is not only capable of improving anti-viral immune responses, but also preventing over-stimulation of the immune system. In fact, immune regulation by CTLA-4 might represent a second built-in safety mechanism next to GITR crucial for preventing over-activation of the triggered immune responses. This would be in consistence with our data showing that CTLA-4^+^ ILC1s display enhanced GITR expression at later time points. In conclusion, the obtained data suggest ILC1 modulation as a valid approach for the establishment of immune interventions against viral infections affecting mucosal areas.

## Ethics Statement

Mice were treated in consensus with local and European Community guidelines and were housed under specific pathogen free conditions in individual ventilated cages with food and water *ad libitum*. The performed animal experiments were approved by the local government in Braunschweig, Germany under the animal permission codes 33.42502-04-13/1281 and 33.19-42502-04-16/2280.

## Author Contributions

NV, PR, ST, TE, and BC designed the experiments. NV and ST performed experiments and acquired data. NV, PR, ST, and BC analyzed data. TE provided reagents. NV, ST, and PR wrote the manuscript draft. CG, ST, and PR discussed data and revised the final manuscript. All authors reviewed and edited the manuscript.

### Conflict of Interest Statement

CG and TE are named as inventors in a patent application covering the use of αGalCerMPEG as adjuvant (EP 05 022 771.9). This does not alter our adherence to the Frontier Immunology policies on sharing data. The remaining authors declare that the research was conducted in the absence of any commercial or financial relationships that could be construed as a potential conflict of interest.
