# Supplementary material for: Invariant NKT Cell-Mediated Modulation of ILC1s as a Tool for Mucosal Immune Intervention
Source: Front Immunol. 2019 Aug 7;10:1849. doi: 10.3389/fimmu.2019.01849 (PMC6692890; doi:10.3389/fimmu.2019.01849)
Supplement: Supplementary file 1 [file Data_Sheet_1.pdf]

SUPPLEMENTARY MATERIAL

Figure S1

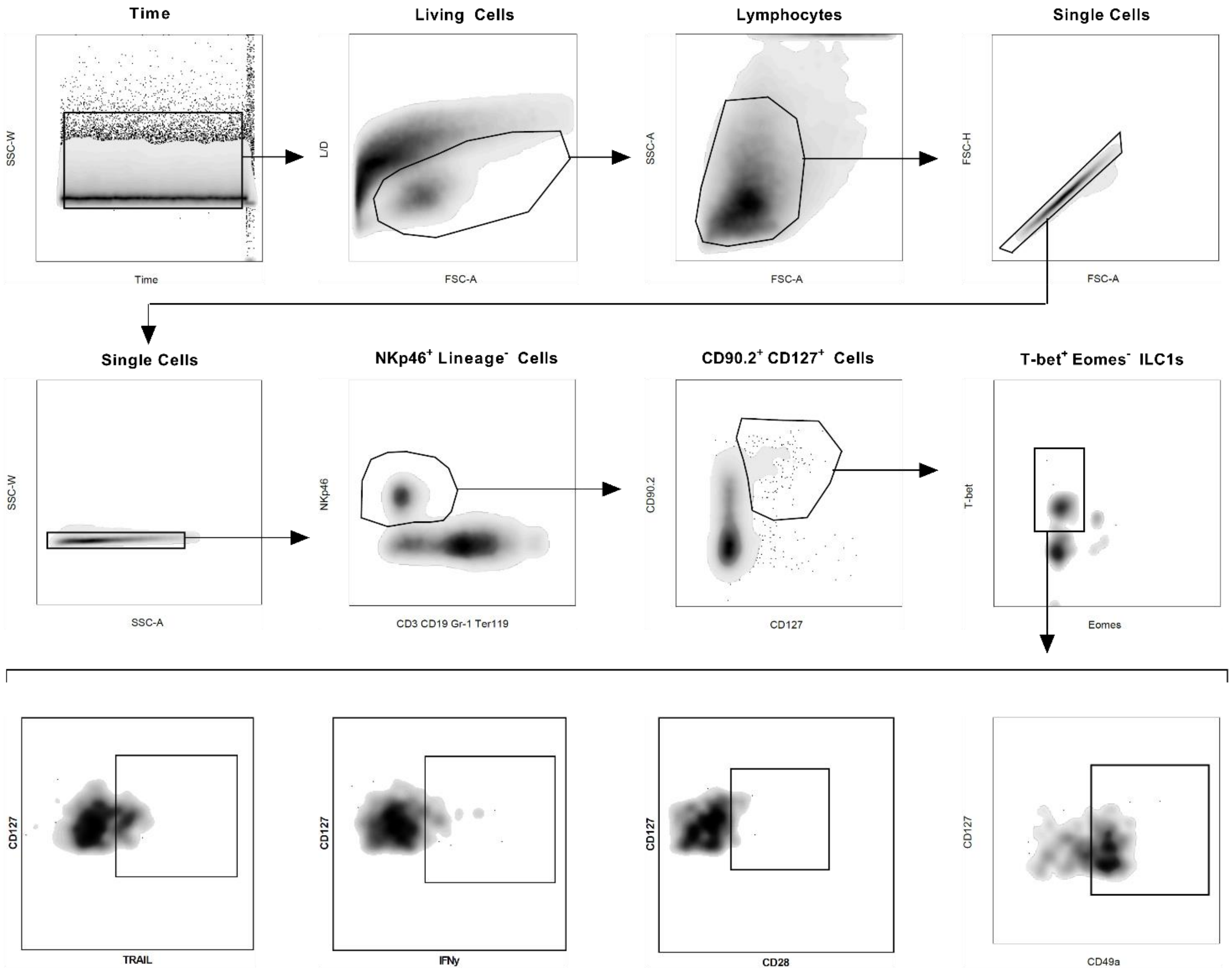

Figure S1: **Flow cytometry gating strategy for the characterization of ILC1s.** For the flow cytometric analysis, cells were gated on Time vs SSC-W to exclude measurement inconsistencies. Subsequently, living singlet lymphocytes were selected and ILC1s were identified as NKp46^{+} Lineage^{-} CD90.2^{+} CD127^{+} T-bet^{+} Eomes^{-} cells.

Figure S2

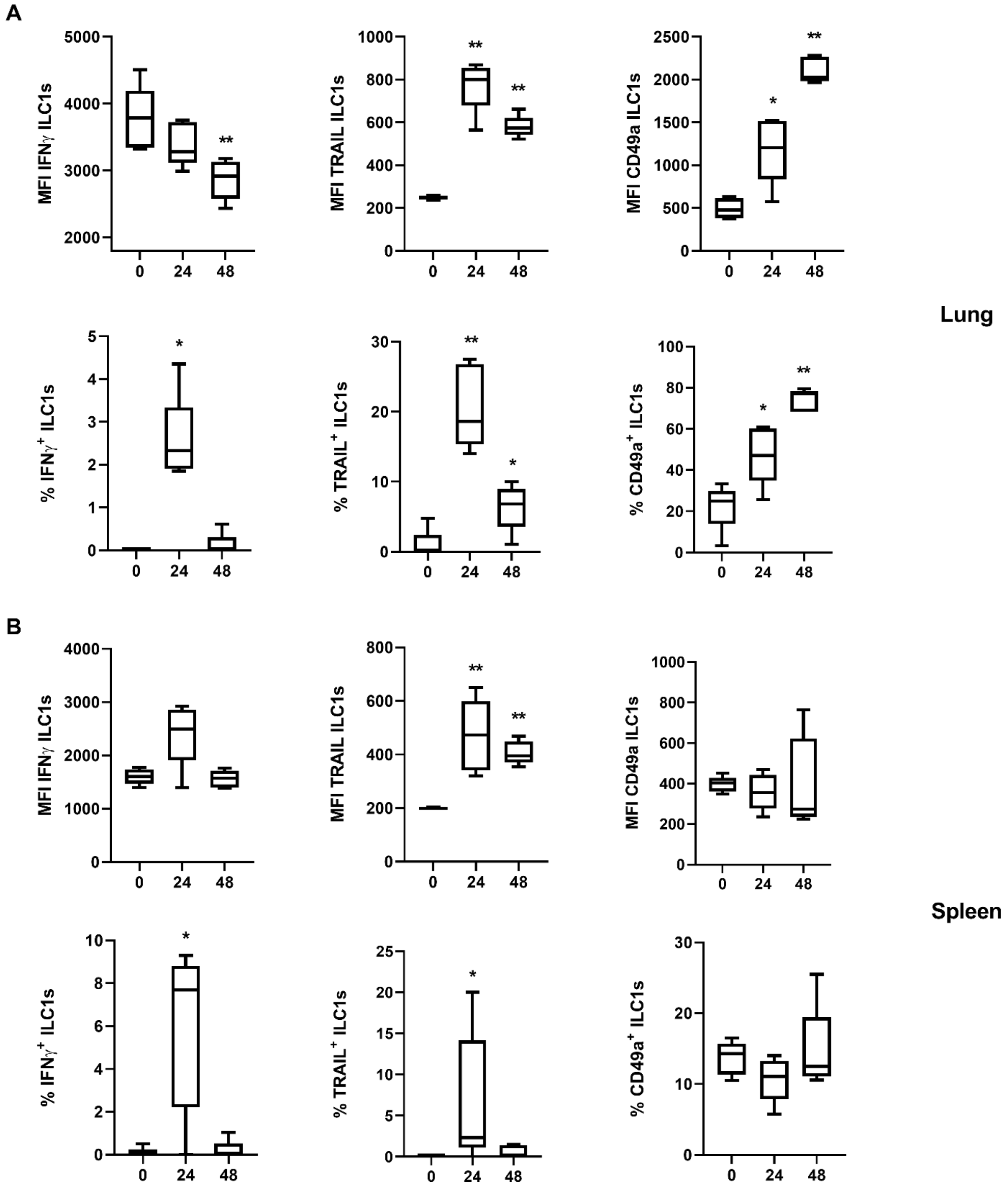

Figure S2: Impact of s.c. administration of αGalCerMPEG on lung and splenic ILC1s. Wild type mice were injected s.c. with a single dose of αGalCerMPEG (5 µg). Lung and splenic lymphocytes were isolated after 0, 24 and 48 h and analyzed for the secretion of IFNγ and the expression of TRAIL and CD49a by flow cytometry following 3 h incubation in the presence of

monensin and brefeldin A. MFI and frequencies (of parent) of ILC1s detected in (A) lung and (B) spleen samples. Box plots represent the range in MFI and frequency variation with the horizontal line indicating the mean. The shown data are derived from one experiment (n= 5). Asterisks denote significant values calculated by Mann-Whitney test; ** $p \leq 0.01$ 1; *$p \leq 0.05$.

Figure S3

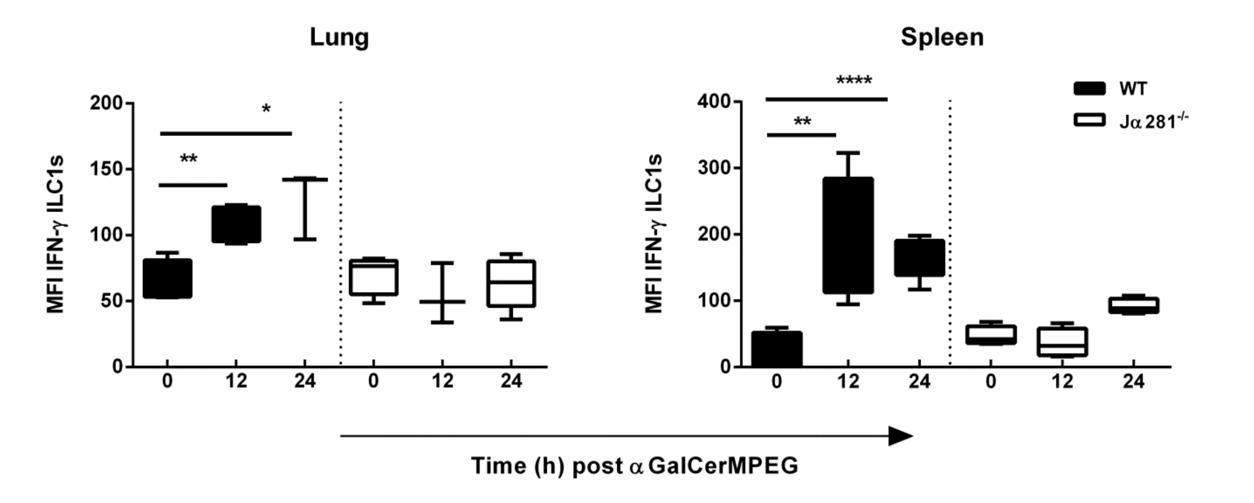

Figure S3: αGalCerMPEG-induced IFNγ secretion by ILC1s is dependent on iNKT cells. Wild type mice and Jα281$^{-/-}$ mice were treated i.n. with a single dose of αGalCerMPEG (5 µg). Lung and splenic lymphocytes were isolated and analyzed for the secretion of IFNγ by flow cytometry following 3 h incubation in the presence of monensin and brefeldin A. MFI IFNγ producing ILC1s derived from lungs and spleens of wild type and Jα281$^{-/-}$ mice. Box plots represent the range in MFI and frequency variation with the horizontal line indicating the mean. The shown data are from one experiment (n= 4-6). Asterisks denote significant values calculated by One-way ANOVA; ****$p \leq 0.0001$; *** $p \leq 0.001$; ** $p \leq 0.01$ 1; *$p \leq 0.05$.

Figure S4

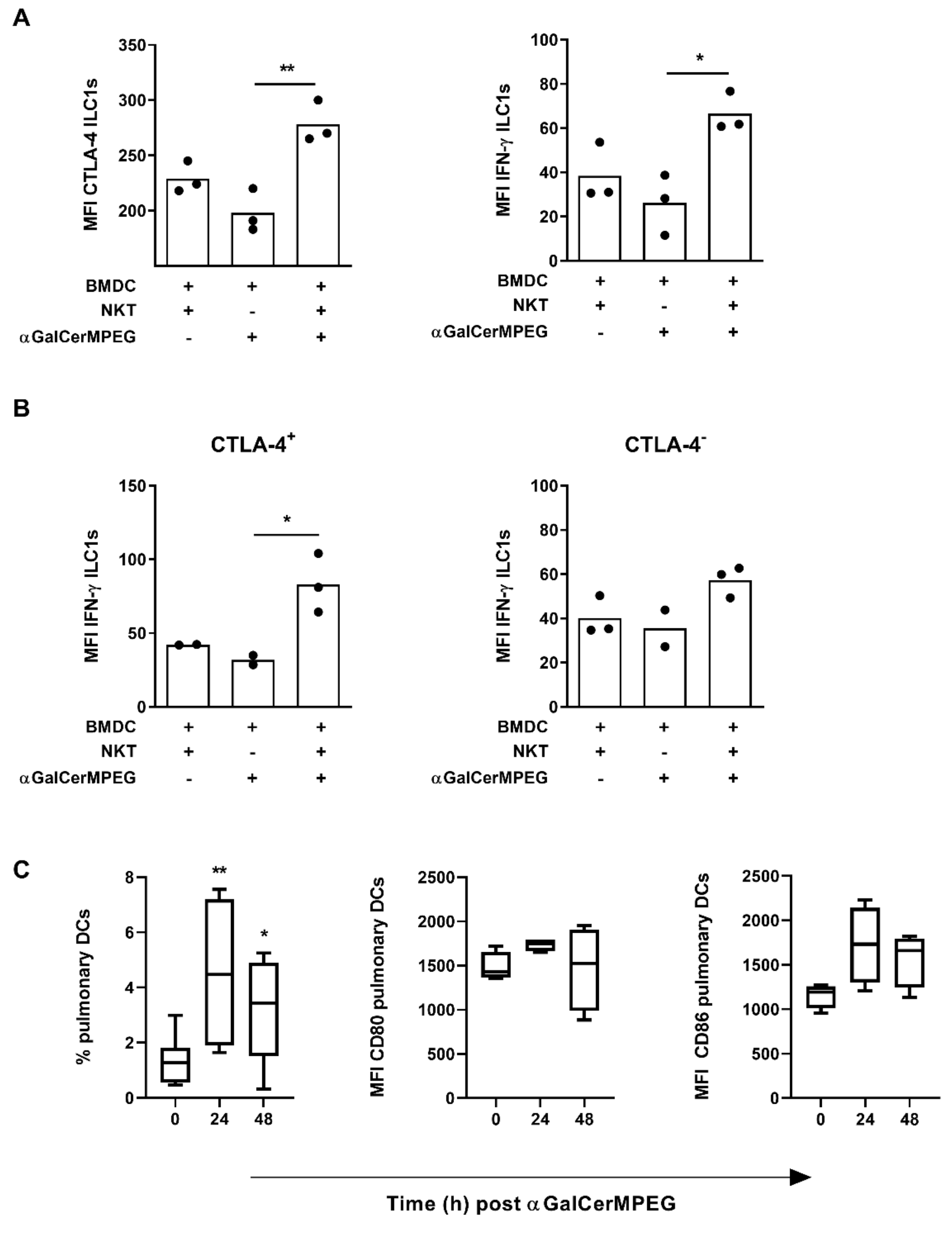

Figure S4: Increased expression of IFNγ from ILC1s following *in vitro* αGalCerMPEG stimulation associates with CTLA-4 expression & impact of αGalCerMPEG administration on pulmonary DCs *in vivo*. FLT-3L differentiated BMDCs from wild type mice were treated with αGalCerMPEG (300 ng/ml) and cultured overnight with *in vitro*-generated ILC1s and sorted splenic NKT cells at a 6:6:1 ratio. Expression of CTLA-4 and secretion of IFNγ were analyzed by flow cytometry following 3 h incubation in the presence of monensin and brefeldin A. (A) MFI of CTLA-4 and IFNγ expressed by ILC1s. (B) MFI of IFNγ production by CTLA-4$^+$ and CTLA-4$^-$ ILC1s. Bars represent mean with single values depicted as dots. MFI data from one experiment are

shown (n=3-4 technical replicates). For the analysis of lung DCs, WT mice were administered i.n. with a single dose of αGalCerMPEG (5 μg). Lungs were harvested at the indicated time points and single cell suspensions were stained for the identification of DCs (MHC cl. II^+ $CD11c^+$) and the expression of CD80 and CD86. (C) Frequencies (of living singlet cells) of DCs and MFI of CD80 and CD86. Box plots represent the range in MFI with the horizontal line indicating the mean. Asterisks denote significant values calculated by One-way ANOVA and Mann-Whitney test; ** $p \leq 0.01$; *$p \leq 0.05$; n.s.

Figure S5

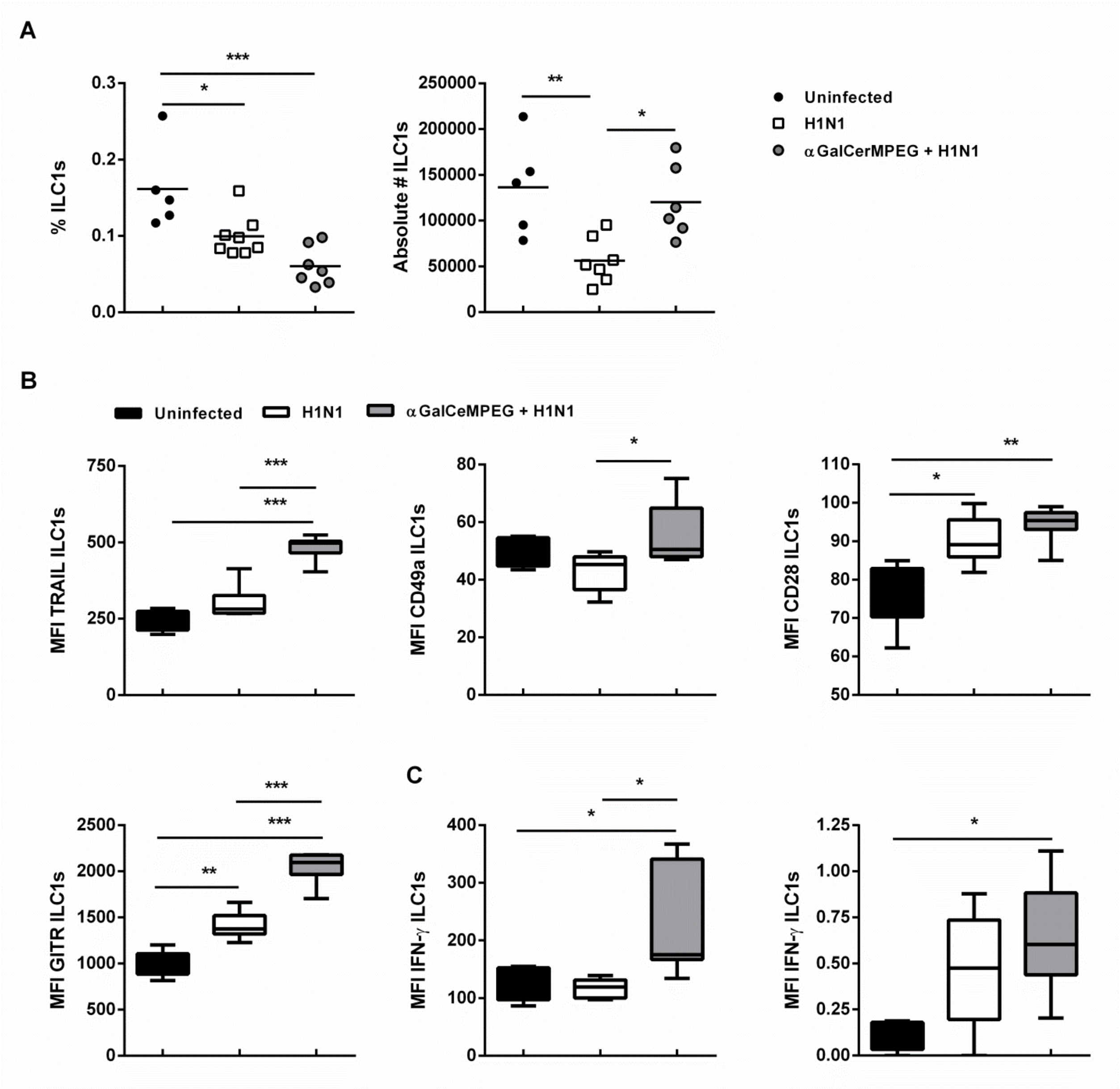

Figure S5: H1N1 infection of αGalCerMPEG-treated mice induced ILC1 activation. Wild type mice were administered i.n. with a single dose of αGalCerMPEG (5 µg) 12 h prior to H1N1 infection ($2*10^3$ ffu). Splenic lymphocytes were isolated 3 days post infection and analyzed for (A) frequencies and absolute numbers of ILC1s and (B) their expression density (MFI) of the activation markers TRAIL, CD49a, CD28 and GITR by flow cytometry analysis. Box plots represent the range in frequency variation as well as absolute cell number with the horizontal line indicating the mean. Frequency and absolute cell number data are representative from one out of two independent experiments (each with n= 5-7). MFI data for the expression of the activation markers are shown from one experiment (n= 5-7). (C) Splenic lymphocytes secreting IFNγ (MFI and frequencies) analyzed by flow cytometry following 3 h incubation in media with monensin and brefeldin A. MFI and frequency data are representative from one out of two independent experiments (each

with n= 5-7). Asterisks denote significant values calculated by One-way ANOVA; *** $p \leq 0.001$; ** $p \leq 0.01$; *$p \leq 0.05$.
